# A68 CROSSING PATHS: THE OVERLAP BETWEEN INTESTINAL TB AND CROHN’S DISEASE, A CASE REPORT

**DOI:** 10.1093/jcag/gwae059.068

**Published:** 2025-02-10

**Authors:** D Denier, J O’Donnell, A Griffiths, T Walters

**Affiliations:** Division of Gastroenterology, Hepatology and Nutrition, The Hospital for Sick Children (SickKids), Toronto, ON, Canada; Division of Gastroenterology, Hepatology and Nutrition, The Hospital for Sick Children (SickKids), Toronto, ON, Canada; Division of Gastroenterology, Hepatology and Nutrition, The Hospital for Sick Children (SickKids), Toronto, ON, Canada; Division of Gastroenterology, Hepatology and Nutrition, The Hospital for Sick Children (SickKids), Toronto, ON, Canada

## Abstract

**Background:**

We report the case of a 17-year-old female, who initially presented with a 3-week history of abdominal pain to a hospital in India, an area endemic for tuberculosis (TB). An abdominal CT scan and endoscopy showed circumferential narrowing involving the cecum and terminal ileum (TI). A tuberculin skin test was positive, while PCR from biopsies was negative. The differential diagnosis was Intestinal TB versus Ileocecal Crohn’s disease (CD). Empirical TB treatment was started, resulting in rapid symptom improvement. She presented to our hospital after immigration with increasing abdominal pain, vomiting and findings of a short, narrowed segment of TI on ultrasound. The patient developed two subsequent episodes of complete bowel obstruction, prompting urgent IC resection. At operation there was a stricture at the level of the cecum, the TI was noted to be thickened with proximal dilatation. Pathology showed inconclusive results with acute and chronic inflammation, possibly inflammatory bowel disease (IBD) or infectious etiology. Repeat quantiferon test was positive with high titre.

**Aims:**

Abdominal TB can uncommonly cause intestinal stricturing during the active phase of disease. The potential for a very similar presentation to CD makes differentiation challenging. In this case report we discuss the background and potential approaches to stricturing intestinal disease in patients from a TB endemic area and the implications for post-operative management.

**Methods:**

Case report with literature review

**Results:**

Gastrointestinal TB can occur as a primary infection or in the context of active pulmonary disease. TB PCR is highly specific but with low sensitivity and Quantiferon Test may be negative in patients with extra pulmonary manifestations. Differentiating between intestinal TB and CD is challenging. In TB endemic areas, the standard approach is to treat for intestinal TB first, only considering CD if symptoms persist; a markedly different approach than practiced in Canada.

Our patient primarily presented with intestinal obstruction showing initial improvement after anti-TB treatment, followed by rapid evolution of fibrostenosis. Confirmation of TB diagnosis was complicated by limited evaluation prior to anti-TB therapy and inconclusive test results subsequently. Although surgical resection was inevitable in this case, post-operative medical management becomes problematic in the absence of a confirmed diagnosis where the choices include ongoing anti-TB therapy versus the initiation of CD post-operative prophylaxis with anti-TNF therapy.

**Conclusions:**

Clinicians should be aware that intestinal TB can cause intestinal stenosis, especially in the IC region. Diagnostic clarity should be top priority, in order to provide the best possible long-term outcomes.

**Funding Agencies:**

**None**

